# MXenes in Cancer Nanotheranostics

**DOI:** 10.3390/nano12193360

**Published:** 2022-09-27

**Authors:** Siavash Iravani, Rajender S. Varma

**Affiliations:** 1 Faculty of Pharmacy and Pharmaceutical Sciences, Isfahan University of Medical Sciences, Isfahan 81746-73461, Iran; 2 Regional Centre of Advanced Technologies and Materials, Czech Advanced Technology and Research Institute, Palacký University in Olomouc, Šlechtitelů 27, 783 71 Olomouc, Czech Republic

**Keywords:** MXenes, MXene-based composites, nanotheranostics, cancer diagnostics, cancer therapeutics

## Abstract

MXenes encompass attractive properties such as a large surface area, unique chemical structures, stability, elastic mechanical strength, excellent electrical conductivity, hydrophilicity, and ease of surface functionalization/modifications, which make them one of the broadly explored two-dimensional materials in the world. MXene-based micro- and nanocomposites/systems with special optical, mechanical, electronic, and excellent targeting/selectivity features have been explored for cancer nanotheranostics. These materials exhibit great diagnostic and therapeutic potential and offer opportunities for cancer photoacoustic imaging along with photodynamic and photothermal therapy. They can be applied to targeted anticancer drug delivery while being deployed for the imaging/diagnosis of tumors/cancers and malignancies. MXene-based systems functionalized with suitable biocompatible or bioactive agents have suitable cellular uptake features with transferring potential from vascular endothelial cells and specific localization, high stability, and auto-fluorescence benefits at different emission–excitation wavelengths, permitting post-transport examination and tracking. The surface engineering of MXenes can improve their biocompatibility, targeting, bioavailability, and biodegradability along with their optical, mechanical, and electrochemical features to develop multifunctional systems with cancer theranostic applications. However, challenges still persist in terms of their environmentally benign fabrication, up-scalability, functionality improvement, optimization conditions, surface functionalization, biocompatibility, biodegradability, clinical translational studies, and pharmacokinetics. This manuscript delineates the recent advancements, opportunities, and important challenges pertaining to the cancer nanotheranostic potential of MXenes and their derivatives.

## 1. Introduction

Today, designing multifunctional systems with unique mechanical, photothermal, and optical characteristics for simultaneous treatment and diagnosis is very helpful for faster diagnosis and recovery as well as better monitoring of the therapeutic process of the patient [1,2,3]. Typically, chemotherapy, hormonal therapy, surgery, radiotherapy, and immunotherapy have been widely applied in the treatment of cancers or elimination of tumors [4,5]. However, these routes may suffer from some limitations/challenges such as low selectivity/sensitivity, poor drug bioavailability, low biocompatibility, high toxicity, off-target effects, high dose necessities, multidrug resistance, etc., which has prompted researchers to envision and explore effective alternative strategies or systems with high efficiency and specific targeting properties to reduce possible off-target effects and improve multifunctionality [6,7,8,9]. Designing two-dimensional (2D) nanostructures from graphene and its derivatives, black phosphorus, graphitic carbon nitride, MXenes, and transition metal dichalcogenides for simultaneous cancer therapy and diagnosis is one of them [10,11,12,13]. Overall, 2D nanostructures have shown the appealing advantages of more tumbling and rolling dynamics during flow in the blood compared to other nanomaterials, providing significant enhancement in the lateral accumulation via the vessels in tumors [14,15,16,17,18,19]. The optical and thermodynamic properties of 2D nanostructures can be organized by controlling the number of atomic layers, defect sites, dimensions, or decoration of other plasma materials; these materials with a large specific surface area can be applied to the loading and targeted delivery of chemotherapy drugs, photosensitizers, and immune adjuvants, among others [20,21].

MXenes with the general formula of M_n+1_X_n_T_x_ and abundance of functionalities (such as –O, –OH, –Cl, and –F) are ideally suited for surface functionalization or modification, thus paving the way for designing smart micro- and nanosystems with multifunctionality. In general, several strategies have been introduced for surface functionalization of MXenes based on covalent and noncovalent modification processes [22,23,24]. The noncovalent surface modification can be achieved via the combination of van der Waals forces, hydrogen bonding, and electrostatic attraction [25]. On the other hand, covalent surface functionalization techniques are based on the application of small molecules (e.g., epoxy compounds, organic amines, acid anhydrides, alkali metal hydroxides, and acid halides) and surface-initiated polymerization by macromolecules as well as single heteroatom methods [26]. MXenes have shown excellent capabilities for synergistic treatment, encompassing chemotherapeutic drugs, photosensitizers, and immune adjuvants [27]. Compared to other 2D structures such as graphene, transition metal dichalcogenides, black phosphorus nanosheets, metal–organic framework nanosheets, and hexagonal boron nitride, MXenes have a low density, excellent electrical conductivity, hydrophilicity, unique optical/thermal properties, and biocompatibility. They exhibit the advantages of magnetic properties and tunable electric features, making them promising candidates for cancer nanotheranostics [28]. Although graphene displays high electrical conductivity, it has low grade magnetic properties. Therefore, it cannot be applied to electromagnetic interference shielding applications as adequate magnetic dipoles where conductivity for interacting with electromagnetic waves is essential. However, several hydrogels combined with conductive functional materials were introduced with unique mechanical flexibility, fatigue durability, and suitable stretchability, which can be further utilized in designing flexible functional devices [29,30]. In addition, tunable conductivity by MXenes ranges from metallic to semiconductor; these structures can be fabricated using cost-effective and simple techniques. However, designing multifunctional MXene-based systems with controllable properties and high stability is an important challenging issue [31,32]. In this context, the long-term toxicity of pristine and surface-modified MXenes ought to be systematically evaluated on humans and the environment [23]. 

**Figure 1:**
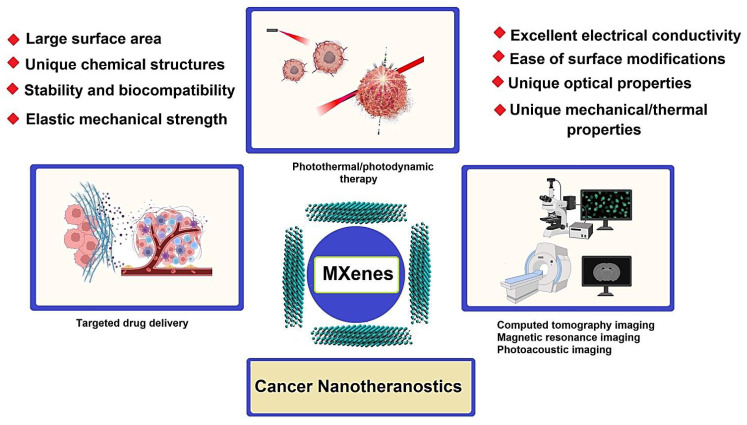
MXenes with cancer nanotheranostic applications.

Recently, MXenes and their composites have garnered much attention in cancer nanotheranostics due to their fascinating mechanical, optical, electronic, and thermal features [27,33,34,35,36,37,38]; their hydrophilicity and high surface area for functionalization/modification make them promising candidates for targeted cancer nanotherapy along with specific imaging/diagnosis of cancer cells/tumor sites (Figure 1 and Table 1) [12,39,40,41,42]. Advanced MXene-based systems with the benefits of improved solubility, high targeting/selectivity properties, multifunctionality, biocompatibility, and low toxicity have shown suitability for targeted anticancer drug delivery, and photothermal, photodynamic, and chemodynamic therapy along with magnetic resonance and computed tomography imaging [27,43,44,45,46]. Zhu et al. [47] demonstrated that when MXenes (Ti_3_C_2_) nanosheets with superb near-infrared (NIR) responsiveness were combined with gold nanorods, nanohybrids with excellent photothermal conversion efficiency could be obtained for cancer therapy owing to the notable photothermal synergy between the gold nanorods and MXenes. Additionally, MXenes can be applied to load anticancer drugs (doxorubicin) with distinct pH/NIR responsive drug release behaviors upon NIR irradiation, owing to the strong π-π stacking interaction between the MXene-based composites and doxorubicin [47]. In addition, MXenes with a high drug-loading capacity and photothermal conversion capability exhibited pH-responsive and NIR laser-stimulated on-demand drug release behaviors, thus opening a new window for synergistic photothermal tumor ablation and chemotherapy (in vitro and in vivo) [48,49]. These materials can be employed as contrast agents for photoacoustic imaging, offering excellent potential for diagnostic imaging guidance and monitoring during the therapeutic process [50,51]. Herein, the most recent developments in cancer nanotheranostic applications of MXenes and their composites are deliberated. 

## 2. MXenes with Cancer Nanotheranostic Potential

Several studies have focused on the design of MXenes and their composites with diagnostic and therapeutic potential [65,66,67,68,69,70,71]. However, compared to other evaluated 2D structures such as graphene and its derivatives, limited studies have been devoted to the simultaneous therapeutic and diagnostic use of these structures thus far [59,72,73]. MXenes with their unique architectures and surface chemistry specifically for the in situ growth of superparamagnetic Fe_3_O_4_ nanocrystals were applied in the design of superparamagnetic 2D MXene (Ti_3_C_2_)-based structures for precise cancer theranostic applications [53]. These biocompatible composites exhibited a high photothermal conversion efficiency (~48.6%) for the photothermal elimination of cancer cells and ablation of tumor tissues with high efficiency (in vitro and in vivo) along with excellent T_2_ relaxivity (~394.2 mM^−1^ s^−1^) and efficient contrast-enhanced MRI of tumors, paving a new pathway for cancer theranostics [53]. Similarly, ultrathin Ta_4_C_3_ MXene nanosheets were synthesized and utilized for in situ growth of superparamagnetic iron oxide nanomaterials onto their surfaces [61]. These biocompatible composites were further functionalized with soybean phospholipid to improve their stability in physiological conditions. They can be employed for photothermal therapy (with the photothermal conversion efficiency of ~32.5%) as well as contrast-enhanced CT and T_2_-weighted MRI of breast tumors with a high performance, offering promising platforms for cancer theranostics [61]. 

The salient advantages of hydrophilicity and low cytotoxicity make MXene-based structures promising candidates for cancer therapy and diagnosis with biosafety and clinical translation potential [74,75,76]. In addition, these materials, with their broad and strong absorbance in the NIR region, along with their significant light-to-heat conversion efficiency, should be further explored for photoacoustic imaging and photothermal therapy [77,78]. Notably, MXenes exhibited great surface-engineering capabilities due to the abundant oxygen-containing groups on their surfaces, thus enhancing their colloidal stability and prolonging in vivo blood circulation [36]. Despite all these advantages, a large number of them have not been investigated for their biomedical applicability, and most explorations have centered around a few examples such as Ti_3_C_2_, Nb_2_C, Mo_2_C, V_2_C, and Ta_3_C_4_ [52,79]. Designing novel MXene-based structures with multiple theranostic applicability, high biocompatibility, and rapid biodegradation can almost guarantee their multipurpose biomedical application and clinical translation [80]. In addition, hyperthermia-amplified nanozyme catalytic therapy using MXenes can be considered as an alternative strategy for the treatment of cancers [57]. In this context, MXene nanosheets could be employed as substrates to anchor functional components such as nanozymes and nanodrugs. For instance, platinum (Pt) artificial nanozymes were decorated on the surface of MXene (Ti_3_C_2_) nanosheets to obtain nanocomposites with peroxidase-like performance, which could, in situ, catalyze hydrogen peroxide to form hydroxyl radicals (^•^OH) to stimulate cell apoptosis and necrosis. These composites exhibited suitable photothermal effects upon NIR-II light irradiation with a low power density, offering new opportunities for synergistic photothermal/enzyme cancer along with photoacoustic imaging capabilities to guide the therapeutic procedure [57]. 

**Figure 2:**
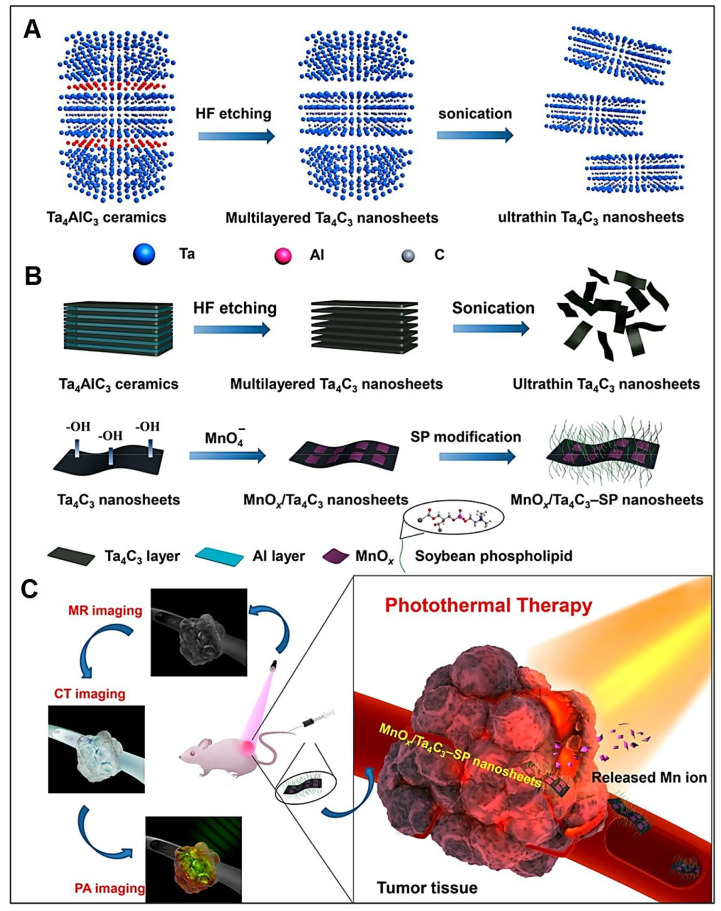
(**A**,**B**) The preparative process of MXene nanosheets including hydrogen fluoride (HF) etching and sonication and their surface functionalization/modification using MnO_x_ and soybean phospholipid (SP). (**C**) MXene-based nanocomposites with photoacoustic (PA), MR, and CT imaging capabilities combined with photothermal effects for tumor ablation. Adapted from Reference [60] with permission. Copyright: 2017, American Chemical Society.

Zong et al. [54] reported the utilization of GdW_10_ nanoclusters (as the contrast agents) for the surface engineering of MXene (Ti_3_C_2_) nanosheets with tumor photothermal therapy and dual MR/CT imaging capabilities (in vivo). These composites with high biocompatibility ought to be further explored for multipurpose nanotheranostics, especially for targeted cancer therapy and diagnosis [54]. In addition, biocompatible MXene (Ti_3_C_2_)-based composites (MnO_x_/Ti_3_C_2_) were introduced for cancer theranostics, providing efficient nanoplatforms for photothermal cancer/tumor nanotherapy with significant tumor ablation and tumor growth suppression effects guided by MR and photoacoustic imaging [55]. In this context, photoacoustic imaging with its advantages of high resolution and contrast in real time and at long penetration depths ought to be further explored using MXenes and their composites owing to their unique optical properties and their excellent potential in photoacoustic imaging for tissue visualization [81]. Dai et al. [60] modified the surface of MXenes (Ta_4_C_3_) utilizing manganese oxide nanoparticles (MnO_x_) for imaging-guided photothermal tumor ablation (Figure 2). Accordingly, the tantalum components of these composites could act as contrast agents with high performance or contrast-enhanced CT, while the incorporated MnO_x_ component performed as the tumor microenvironment-responsive contrast agent for the T_1_-weighted MRI. These composites with high photothermal conversion efficiency could be employed for contrast-enhanced photoacoustic imaging along with the superb growth suppression of tumors via photothermal hyperthermia, providing a biocompatible MXene-based platform for cancer nanotheranostics [60]. 

**Figure 3:**
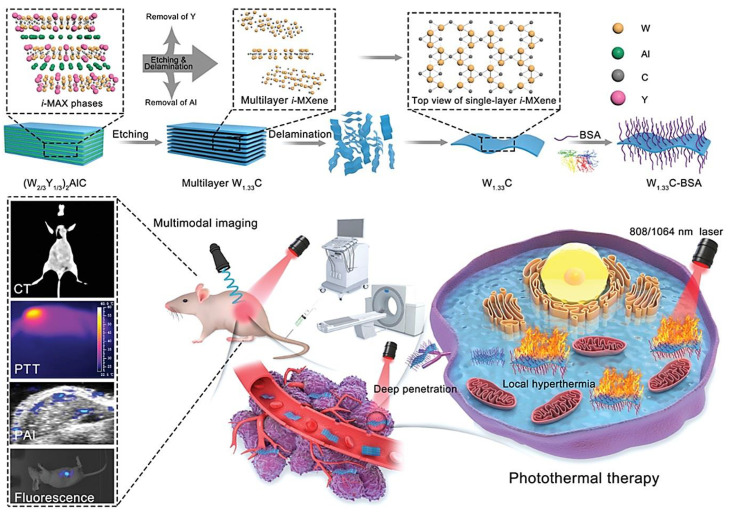
The preparative process of bovine serum albumin (BSA)-modified W_1_._33_*C i*-MXene with high photothermal conversion efficacy for theranostic applications (multimodal imaging and photothermal therapy). CT: computed tomography; PTT: photothermal therapy; and PAI: photoacoustic imaging. Adapted from Reference [77] with permission (CC BY). Copyright: 2021, Wiley-VCH GmbH.

A distinct W_1_._33_C *i*-MXene was developed for theranostic applications with the advantages of rapid biodegradation (in normal tissue rather than in tumors) and improved biocompatibility (Figure 3) [77]. These MXene nanosheets exhibited an excellent predominance of NIR absorbance along with high photothermal conversion effectiveness (~32.5% at NIR-I and ~49.3% at NIR-II); they could be applied as suitable platforms with multimodal-imaging features (suitable for CT and photoacoustic imaging) and photothermal-ablation effects against tumors (in vitro and in vivo). The underlying mechanisms ought to be comprehensively explored using genomics and proteomics [77]. In addition, functionalized MXene-based structures were constructed to obtain distinct tumor microenvironment-responsive T_1_ and T_2_ MRI-guided photothermal breast cancer hyperthermia in the NIR-II bio-window, providing nanoplatforms for the imaging-guided photonic hyperthermia of breast cancers [82]. Accordingly, superparamagnetic Fe_3_O_4_ and paramagnetic MnO_x_ nanomaterials were grown onto the large surface of ultrathin MXene (Nb_2_C) nanosheets. These composites with significant photothermal conversion efficiency in the NIR-II bio-window and suitable biocompatibility could be applied for photothermal tumor suppression [82]. Several MXene-based systems were introduced for synergistic cancer therapy, and their capabilities can be extended with the addition of imaging/diagnostic ability. Liu et al. [83] introduced ultrathin MXene (Ti_3_C_2_) nanosheets (~100 nm) for targeted photothermal/photodynamic/chemo synergistic tumor therapy. These nanomaterials with good in vitro/in vivo biocompatibility demonstrated a superb mass extinction coefficient (~28.6 Lg^−1^ cm^−1^ at 808 nm), high photothermal conversion efficiency (~58.3%), and effective singlet oxygen generation (^1^O_2_) upon 808 nm laser irradiation. In addition, after layer-by-layer surface modification, these multifunctional nanoplatforms could be deployed for targeted delivery of the doxorubicin anticancer drug [83]. It appears that the next step ought to focus on improving their biocompatibility and theranostic application. 

**Figure 4:**
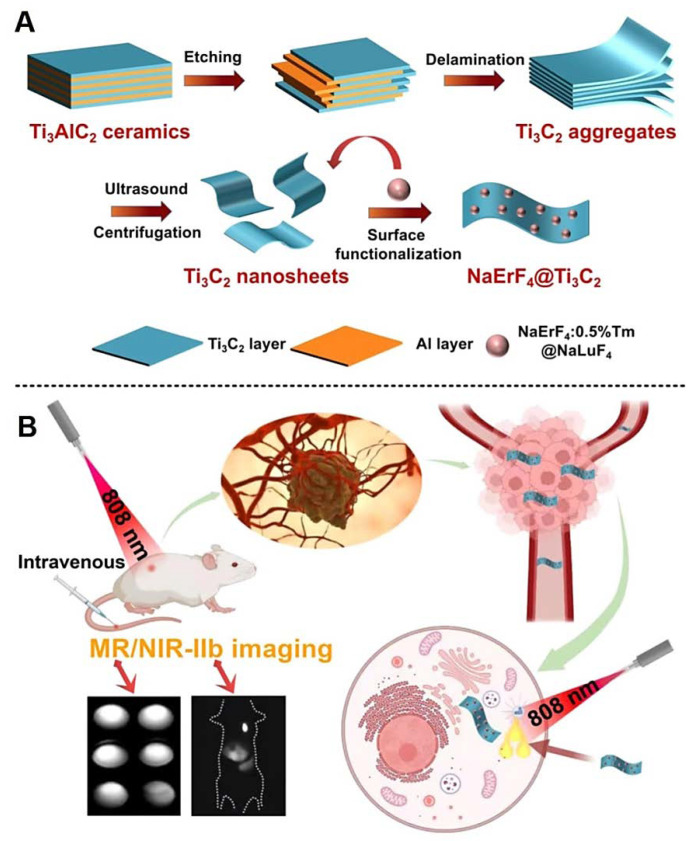
(**A**) The preparative process of NaErF_4_@Ti_3_C_2_ MXene-based nanosystems for cancer theranostic applications. (**B**) Photothermal therapy and MR/NIR-II b imaging of cancer/tumor using the MXene nanocomposites. Adapted from Reference [56] with permission. Copyright: 2022, American Chemical Society.

Mo_2_C MXene nanospheres (~50 nm) were fabricated as theranostic agents, wherein their light harvesting covered the total NIR region. In addition, hyperthermia and reactive oxygen species (ROS) generation can be simultaneously triggered by NIR irradiation [58]. These nanospheres with excellent biocompatibility could be deployed for synergistic photothermal and photodynamic cancer therapy, thus eliminating cancer cells and removing solid tumors (by the typical liquefactive necrosis procedure). They additionally demonstrated suitable photoacoustic and CT imaging applicability (in vivo) [58]. In another study, nanosheets of MXene (Ti_3_C_2_) were functionalized with NaErF_4_ nanoparticles to develop multifunctional platforms for NIR-IIb (1530 nm) and MRI-guided photothermal cancer nanotherapy under 808 nm excitation, providing tumor ablation with an inhibition ratio of ~92.9% (Figure 4) [56]. These nanocomposites, with excellent photothermal conversion potential (43.62% at 808 nm irradiation) and photothermal stability, could be efficiently applied to T_2_-weighted MRI due to the inherent magnetic features of Er^3+^ ions; interestingly, no noticeable toxicity could be detected at the injected dose [56]. Zhang et al. [24] reported the construction of photo/sono-responsive antitumor theranostic nanoplatforms via the decoration of the TiO_2−x_ nanoparticle (~10 nm) on the surface of MXenes (Ti_3_C_2_) for photoacoustic/photothermal bimodal imaging-guided NIR-II photothermal enhanced sonodynamic therapy of tumors. These nanocomposites unveiled enhanced sonodynamic ROS formation along with induced extensive localized hyperthermia, showing excellent tumor eradication (in vivo), with no tumor recurrence and systemic toxicity [24].

MXene-based quantum dots exhibited unique optical features including light absorption, photoluminescence, and electrochemiluminescence, which need to be systematically studied in the field of biomedical engineering, optoelectronic catalysis, and optoelectronics [84]. Among reported MXene-based quantum dots, Ti_2_N quantum dots (~5 nm) with unique photophysical properties displayed high photothermal conversion efficiency under laser irradiation in NIR-I, 808 nm (~48.62%) and NIR-II, 1064 nm (~45.51%) [85]. These quantum dots with high biocompatibility, sufficient stability in circulation, appropriate excretion rate from the body, photoacoustic effects, and photothermal therapy efficiency showed detectable aggregations in tumors after 4 h post-injection and could be deployed for photoacoustic imaging-guided photothermal therapy in NIR-I/II bio-windows with no noticeable toxic effects (in vitro/in vivo) [85]. Such quantum dots can be considered for cancer theranostic applications; future explorations ought to focus on their degradability, biocompatibility, and multifunctionality. 

## 3. Toxicity and Biosafety Issues

Although numerous studies have focused on the applications and synthesis of MXenes and their composites, limited studies have comprehensively explored their toxicity and biosafety aspects, especially in biomedical sciences [86]. Notably, clinical translation studies, along with the industrialization of assigned synthesis techniques, are very important challenges. The crucial factors affecting the toxicity of MXenes ought to be focused on by researchers, including their chemical nature, solubility, size, surface, morphology, aggregation, and their structure [86]. One of most important challenging issues in the translation of theranostic nanomedicines from in vitro to in vivo and then to clinical studies is finding the association between the physicochemical nature of the designed micro- and nanosystems along with their interactions with biological systems [87]. Thus, clinical translational studies are warranted to evaluate the efficiency, off-target toxic effects, and their physicochemical features while they are in circulation. Comprehensive preclinical studies as well as the evaluation of the nanobiological interactions of MXenes and their composites in in vivo systems can help to better identify the limitations and challenges that lie ahead [87]. Another parameter is biodegradability, which is a very important index for analyzing the biosafety of MXenes before their practical application. For instance, it was revealed that MXenes (Nb_2_C) underwent rapid decomposition within 24 h in the presence of human myeloperoxidase and hydrogen peroxide [88]. In addition, ultrathin MXenes (Ti_3_C_2_) exhibited enzymatic and ROS-stimulated biodegradability [89], and MXenes (Mo_2_C) displayed pH-dependent degradation behavior [49]. In contrast, MXenes demonstrated suitable stability in tumor tissues with relatively longer degradation times, offering opportunities for the photothermal ablation of tumors. As the underlying mechanisms of the biodegradability of MXenes have rarely been evaluated, future explorations need to focus on this critical aspect [33,90]. By optimizing the synthesis/reaction and functionalization conditions and avoiding harsh etching/delamination processes, MXenes with good stability can be obtained; ultrathin MXenes with poor chemical stability underwent decomposition through the oxidation reactions. On the other hand, the biodegradation and in vivo bioclearance of MXene-based structures can be accelerated due to the presence of high-strength ionic conditions and abundant enzymes in the physiological environments [33,77].

Several studies have concentrated on the toxicity and biocompatibility of MXenes and their derivatives. In one study, the acute toxicity and histocompatibility evaluations of intravenously administered Ti_3_C_2_-soybean phospholipid nanocomposites exhibited no evidence of pathologies and histomorphological alterations in the examined organs compared to control samples, showing no acute toxicity and adverse effects of these MXenes. In addition, the excretion with urine and feces was ~18.70% and 10.35%, respectively, after 48 h [50]. Furthermore, the biocompatibility and biosafety analyses (in vivo) of the nanocomposites of MnO_x_/Ti_3_C_2_-soybean phospholipid after a single-dose intravenous administration demonstrated that all the major vital signs were normal, indicating no signs of toxic action [55]. Lin et al. [88] studied the toxicity of polyvinyl pyrrolidone-modified MXene (Nb_2_C) nanocomposites with biodegradability attributes as they exhibited no adverse effects on the blood chemistry values. The histological assessments of the heart, liver, spleen, lung and kidney illustrated no pathological alterations in the tissues; the excretion from the body rate and clearance routes indicated that 20% of the niobium was excreted with urine and feces within 48 h, exposing their high biocompatibility [88].

## 4. Conclusions and Perspectives

Although the emergence of MXenes has significantly expanded the family of 2D materials and their versatile applications, the rational design of MXenes and their composites for cancer nanotheranostics with photothermal/photodynamic therapy, radiotherapy, catalytic therapy, and imaging features still remains an important challenge in biomedicine. Hybridization and surface functionalization/modification can help to improve the mechanical, electronic, thermal, and optical properties of these materials for application in cancer therapy and diagnosis. Since clinical translation studies on MXenes and their applications in bio- and nanomedicine are still in their infancy, additional explorations need to focus on the main aspects including optimization conditions, facile and environmentally benign synthesis techniques, clinical translational studies, long-term toxicity/biosafety issues, pharmacokinetics, targeting properties, and their stimuli-responsive manner. Future biosafety investigations are required to comprehensively address the critical issues, including biocompatibility, biodegradability (the degradation rate and degree), blood circulation, and excretion behaviors; the long-term existence of nanomaterials is a serious problem, as it may lead to inflammation, oxidative damage, and fibrosis. On the other hand, limited types of MXenes (mostly Ti_3_C_2_) have recently garnered major interest in biomedical applications owing to their remarkable chemical and physical features. Thus, future cancer theranostic explorations ought to explore other types of MXenes in addition to Ti_3_C_2_ with the careful consideration of optimal conditions/synthesis and functionalization techniques. Notably, MXenes have been applied to photothermal cancer nanotherapy, but their cellular internalization needs to be improved by coating their surfaces with ligands with high specificity towards cancer cells. MXene-based structures with responsiveness to biological triggers (such as pH value, temperature, and enzymes) ought to be innovatively designed for superior therapeutic outcomes.

## Figures and Tables

**Table 1:** Some selected examples of MXenes for cancer nanotheranostics.

MXenes	Applications	Advantages/Benefits	Refs.
Ta_4_C_3_	Dual-mode photoacoustic/computed tomography (CT) imaging along with effective photothermal ablation of tumors (in vivo)	- Excellent photothermal conversion efficiency (η = ~44.7%) - Targeted photothermal ablation of tumors (in vitro and in vivo)	[52]
Ti_3_C_2_	Cancer theranostics; photothermal elimination of cancerous cells and ablation of tumors; magnetic resonance imaging (MRI) of tumors	- Significant T2 relaxivity (~394.2 mM^−1^ s^−1^) with efficient contrast-enhanced MRI - Excellent photothermal conversion efficiency (~48.6%) with high biocompatibility	[53]
Ti_3_C_2_	Photothermal cancer nanotherapy with MR/CT imaging capabilities towards tumor cells or xenografts; applications of GdW_10_@Ti_3_C_2_ nanocomposites as CT contrast agents	- High biocompatibility (in vivo) - Deep penetration and high spatial resolution of CT images - effective accumulation in tumor tissues; high photothermal ablation efficacy (in vivo) - High stability (in vivo) with suitable photothermal conversion efficiency (η = ~21.9%)	[54]
Ti_3_C_2_	MR and photoacoustic dual-modality imaging-guided photothermal cancer therapy	- High biocompatibility (in vivo) - Good photothermal conversion performance (~22.9%) - Efficient tumor ablation and tumor growth suppression	[55]
Ti_3_C_2_	Dual-modal NIR-II/MRI-guided tumor hyperthermia	- Efficient T2-weighted MRI - High photothermal conversion efficiency (~43.62% at 808 nm irradiation) - High photothermal stability and effects - Efficient tumor ablation (the inhibition ratio was ~92.9%)	[56]
Ti_3_C_2_	Photothermal cancer therapy; photoacoustic imaging capabilities	- Suitable photothermal effects upon NIR-II light irradiation with a low power density (0.75 W cm^−2^) - Efficient nanosystems for hyperthermia-amplified nanozyme catalytic therapy	[57]
Mo_2_C	Phototherapy of tumor/cancer using multi-modal imaging-guided strategy	- High biocompatibility - Minimal toxicity and hematotoxicity	[58]
Nb_2_C	Chemo/photothermal cancer therapy; diagnostic potential	- Targeted chemotherapy with reduced toxicity - Improved photothermal hyperthermia of cancer - Low/noncytotoxicity (at 300 μg mL^−1^) - The photothermal conversion efficiency was ~28.6%	[59]
Ta_4_C_3_	Photothermal therapy and photoacoustic imaging of cancers with contrast-enhanced properties	- Excellent growth suppression of tumor - No noticeable toxicity - No noticeable histological defects or lesions in the organs - The efficiency of photothermal conversion was ~34.9%	[60]
Ta_4_C_3_	MRI/CT imaging guided photothermal breast cancer therapy	- Excellent photothermal ablation of breast tumors - No noticeable toxicity with good biocompatibility - The efficiency of photothermal conversion was ~32.5%	[61]
Ti_3_C_2_	MRI/CT imaging guided photothermal cancer therapy	- No noticeable cell necrosis - Low toxicity with good biocompatibility - The efficiency of photothermal conversion was ~21.9%	[62]
V_2_C	MR/photoacoustic guided photothermal cancer treatment	- No noticeable adverse side effects with good biocompatibility - Excellent ablation of tumors - The efficiency of photothermal conversion was ~45.05%	[63]
V2C	MR/photoacoustic guided photothermal cancer treatment	- Low toxicity with good biodistribution - The efficiency of photothermal conversion was ~48 %	[64]
Ti_3_C_2_	Photoacoustic/CT guided photothermal cancer treatment	- High stability with good biocompatibility; - Low long-term toxicity - Core–shell nanocomposites with efficient cancer therapeutic potential	[65]

## Data Availability

Not applicable.

## References

[1] Selestin Raja I., Kang M.S., Kim K.S., Jung Y.J., Han D.-W. (2020). Two-Dimensional Theranostic Nanomaterials in Cancer Treatment: State of the Art and Perspectives. Cancers.

[2] Yang W., Lyu Q., Zhao J., Cao L., Hao Y., Zhang H. (2020). Recent advance in near-infrared/ultrasound-sensitive 2D-nanomaterials for cancer therapeutics. Sci. China Mater..

[3] Huang M., Gu Z., Zhang J., Zhang D., Zhang H., Yang Z., Qu J. (2021). MXene and black phosphorus based 2D nanomaterials in bioimaging and biosensing: Progress and perspectives. J. Mater. Chem. B.

[4] Wan L., Zhao Q., Zhao P., He B., Jiang T., Zhang Q., Wang S. (2014). Versatile hybrid polyethyleneimine–mesoporous carbon nanoparticles for targeted delivery. Carbon.

[5] Senapati S., Mahanta A.K., Kumar S., Maiti P. (2018). Controlled drug delivery vehicles for cancer treatment and their performance. Signal Transduct. Target. Ther..

[6] Chari R.V.J. (2007). Targeted cancer therapy: Conferring specificity to cytotoxic drugs. Acc. Chem. Res..

[7] Murugan C., Sharma V., Murugan R.K., Malaimegu G., Sundaramurthy A. (2019). Two-dimensional cancer theranostic nanomaterials: Synthesis, surface functionalization and applications in photothermal therapy. J. Control. Release.

[8] Shi Z., Zhou Y., Fan T., Lin Y., Zhang H., Mei L. (2020). Inorganic nano-carriers based smart drug delivery systems for tumor therapy. Smart Mater. Med..

[9] Fusco L., Gazzi A., Peng G., Shin Y., Vranic S., Bedognetti D., Vitale F., Yilmazer A., Feng X., Fadeel B. (2020). Graphene and other 2D materials: A multidisciplinary analysis to uncover the hidden potential as cancer theranostics. Theranostics.

[10] Jain V., Jain S., Mahajan S.C. (2015). Nanomedicines based drug delivery systems for anti-cancer targeting and treatment. Curr. Drug Deliv..

[11] Kumar P., Srivastava R. (2016). Nanomedicine for Cancer Therapy: From Chemotherapeutic to Hyperthermia-Based Therapy.

[12] Hiremath N., Kumar R., Hwang K.C., Banerjee I., Thangudu S., Vankayala R. (2022). Near-Infrared Light Activatable Two-Dimensional Nanomaterials for Theranostic Applications: A Comprehensive Review. ACS Appl. Nano Mater..

[13] Korupalli C., You K.-L., Getachew G., Rasal A.S., Dirersa W.B., Fahmi M.Z., Chang J.-Y. (2022). Engineering the Surface of Ti_3_C_2_ MXene Nanosheets for High Stability and Multimodal Anticancer Therapy. Pharmaceutics.

[14] Blanco E., Shen H., Ferrari M. (2015). Principles of nanoparticle design for overcoming biological barriers to drug delivery. Nat. Nanotechnol..

[15] Abbasi Z., Feizi S., Taghipour E., Ghadam P. (2017). Green synthesis of silver nanoparticles using aqueous extract of dried Juglans regia green husk and examination of its biological properties. Green Process. Synth..

[16] Iravani S. (2022). MXenes and MXene-based (nano)structures: A perspective on greener synthesis and biomedical prospects. Ceram. Int..

[17] Iravani S., Varma R.S. (2021). MXenes and MXene-based materials for tissue engineering and regenerative medicine: Recent advances. Mater. Adv..

[18] Iravani S., Varma R.S. (2021). MXenes for Cancer Therapy and Diagnosis: Recent Advances and Current Challenges. ACS Biomater. Sci. Eng..

[19] Iravani S., Varma R.S. (2022). Bioinspired and biomimetic MXene-based structures with fascinating properties: Recent advances. Mater. Adv..

[20] Ni N., Zhang X., Ma Y., Yuan J., Wang D., Ma G., Dong J., Sun X. (2022). Biodegradable two-dimensional nanomaterials for cancer theranostics. Coord. Chem. Rev..

[21] Yang B., Chen Y., Shi J. (2018). Material Chemistry of Two-Dimensional Inorganic Nanosheets in Cancer Theranostics. Chem.

[22] Ibragimova R., Erhart P., Rinke P., Komsa H.-P. (2021). Surface Functionalization of 2D MXenes: Trends in Distribution, Composition, and Electronic Properties. J. Phys. Chem. Lett..

[23] Mozafari M., Soroush M. (2021). Surface functionalization of MXenes. Mater. Adv..

[24] Zhang D.-Y., Liu H., Younis M.R., Lei S., Chen Y., Huang P., Lin J. (2022). In-situ TiO_2−x_ decoration of titanium carbide MXene for photo/sono-responsive antitumor theranostics. J. Nanobiotechnol..

[25] Chitteth Rajan A., Mishra A., Satsangi S., Vaish R., Mizuseki H., Lee K.-R., Singh A.K. (2018). Machine-Learning-Assisted Accurate Band Gap Predictions of Functionalized MXene. Chem. Mater.

[26] Qin L., Tao Q., Liu X., Fahlman M., Halim J., Persson P.O.Å., Rosen J., Zhang F. (2019). Polymer-MXene composite films formed by MXene-facilitated electrochemical polymerization for flexible solid-state microsupercapacitors. Nano Energy.

[27] Dong L.M., Ye C., Zheng L.L., Gao Z.F., Xia F. (2020). Two-dimensional metal carbides and nitrides (MXenes): Preparation, property, and applications in cancer therapy. Nanophotonics.

[28] Shukla V. (2020). The tunable electric and magnetic properties of 2D MXenes and their potential applications. Mater. Adv..

[29] Zeng Z.-H., Wu N., Wei J.-J., Yang Y.-F., Wu T.-T., Li B., Hauser S.B., Yang W.-D., Liu J.-R., Zhao S.-Y. (2022). Porous and Ultra-Flexible Crosslinked MXene/Polyimide Composites for Multifunctional Electromagnetic Interference Shielding. Nano-Micro Lett..

[30] Yang Y., Han M., Liu W., Wu N., Liu J. (2022). Hydrogel-based composites beyond the porous architectures for electromagnetic interference shielding. Nano Res..

[31] Shukla V. (2020). Observation of critical magnetic behavior in 2D carbon based composites. Nanoscale Adv..

[32] Shukla V. (2019). Review of electromagnetic interference shielding materials fabricated by iron ingredients. Nanoscale Adv..

[33] Chen L., Dai X., Feng W., Chen Y. (2022). Biomedical Applications of MXenes: From Nanomedicine to Biomaterials. Acc. Mater. Res..

[34] Sadiq M., Pang L., Johnson M., Sathish V., Zhang Q., Wang D. (2021). 2D Nanomaterial, Ti_3_C_2_ MXene-Based Sensor to Guide Lung Cancer Therapy and Management. Biosensors.

[35] Sharifuzzaman M., Barman S.C., Zahed M.A., Sharma S., Yoon H., Nah J.S., Kim H., Park J.Y. (2020). An Electrodeposited MXene-Ti_3_C_2_T_x_ Nanosheets Functionalized by Task-Specific Ionic Liquid for Simultaneous and Multiplexed Detection of Bladder Cancer Biomarkers. Small.

[36] Sundaram A., Ponraj J.S., Wang C., Peng W.K., Manavalan R.K., Dhanabalan S.C., Zhang H., Gaspar J. (2020). Engineering of 2D transition metal carbides and nitrides MXenes for cancer therapeutics and diagnostics. J. Mater. Chem. B.

[37] Xing C., Chen S., Liang X., Liu Q., Qu M., Zou Q., Li J., Tan H., Liu L., Fan D. (2018). Two-Dimensional MXene (Ti_3_C_2_)-Integrated Cellulose Hydrogels: Toward Smart Three-Dimensional Network Nanoplatforms Exhibiting Light-Induced Swelling and Bimodal Photothermal/Chemotherapy Anticancer Activity. ACS Appl. Mater. Interfaces.

[38] Zamhuri A., Lim G.P., Ma N.L., Tee K.S., Soon C.F. (2021). MXene in the lens of biomedical engineering: Synthesis, applications and future outlook. BioMed Eng. OnLine.

[39] Hendijani F. (2015). Human mesenchymal stromal cell therapy for prevention and recovery of chemo/radiotherapy adverse reactions. Cytotherapy.

[40] Pardoll D.M. (2012). The blockade of immune checkpoints in cancer immunotherapy. Nat. Rev. Cancer.

[41] Jamalipour Soufi G., Iravani S. (2020). Eco-friendly and sustainable synthesis of biocompatible nanomaterials for diagnostic imaging: Current challenges and future perspectives. Green Chem..

[42] Zhu W., Li H., Luo P. (2021). Emerging 2D Nanomaterials for Multimodel Theranostics of Cancer. Front. Bioeng. Biotechnol..

[43] Dong X., Mumper R.J. (2010). Nanomedicinal strategies to treat multidrug-resistant tumors: Current progress. Nanomedicine.

[44] Nasrollahzadeh M., Sajjadi M., Iravani S., Varma R.S. (2020). Trimetallic Nanoparticles: Greener Synthesis and Their Applications. Nanomaterials.

[45] Iravani S., Varma R.S. (2019). Plants and plant-based polymers as scaffolds for tissue engineering. Green Chem..

[46] Iravani S., Varma R.S. (2020). Green synthesis, biomedical and biotechnological applications of carbon and graphene quantum dots. A review. Environ. Chem. Lett..

[47] Zhu B., Shi J., Liu C., Li J., Cao S. (2021). In-situ self-assembly of sandwich-like Ti_3_C_2_ MXene/gold nanorods nanosheets for synergistically enhanced near-infrared responsive drug delivery. Ceram. Int..

[48] Shurbaji S., Abdul Manaph N.P., Ltaief S.M., Al-Shammari A.R., Elzatahry A., Yalcin H.C. (2021). Characterization of MXene as a Cancer Photothermal Agent Under Physiological Conditions. Front. Nanotechnol..

[49] Feng W., Wang R., Zhou Y., Ding L., Gao X., Zhou B., Hu P., Chen Y. (2019). Ultrathin Molybdenum Carbide MXene with Fast Biodegradability for Highly Efficient Theory-Oriented Photonic Tumor Hyperthermia. Adv. Funct. Mater..

[50] Han X., Huang J., Lin H., Wang Z., Li P., Chen Y. (2018). 2D Ultrathin MXene-Based Drug-Delivery Nanoplatform for Synergistic Photothermal Ablation and Chemotherapy of Cancer. Adv. Healthc. Mater..

[51] Mohammadpour Z., Majidzadeh-A K. (2020). Applications of Two-Dimensional Nanomaterials in Breast Cancer Theranostics. ACS Biomater. Sci. Eng..

[52] Lin H., Wang Y., Gao S., Chen Y., Shi J. (2018). Theranostic 2D Tantalum Carbide (MXene). Adv. Mater..

[53] Liu Z., Zhao M., Lin H., Dai C., Ren C., Zhang S., Peng W., Chen Y. (2018). 2D magnetic titanium carbide MXene for cancer theranostics. J. Mater. Chem. B.

[54] Zong L., Wu H., Lin H., Chen Y. (2018). A polyoxometalate-functionalized two-dimensional titanium carbide composite MXene for effective cancer theranostics. Nano Res..

[55] Dai C., Lin H., Xu G., Liu Z., Wu R., Chen Y. (2017). Biocompatible 2D Titanium Carbide (MXenes) Composite Nanosheets for pH-Responsive MRI-Guided Tumor Hyperthermia. Chem. Mater..

[56] Pan J., Zhang M., Fu G., Zhang L., Yu H., Yan X., Liu F., Sun P., Jia X., Liu X. (2022). Ti_3_C_2_ MXene Nanosheets Functionalized with NaErF4:0.5%Tm@NaLuF4 Nanoparticles for Dual-Modal Near-Infrared IIb/Magnetic Resonance Imaging-Guided Tumor Hyperthermia. ACS Appl. Nano Mater..

[57] Zhu Y., Wang Z., Zhao R., Zhou Y., Feng L., Gai S., Yang P. (2022). Pt Decorated Ti_3_C_2_T_x_ MXene with NIR-II Light Amplified Nanozyme Catalytic Activity for Efficient Phototheranostics. ACS Nano.

[58] Zhang Q., Huang W., Yang C., Wang F., Song C., Gao Y., Qiu Y., Yan M., Yang B., Guo C. (2019). The theranostic nanoagent Mo2C for multi-modal imaging-guided cancer synergistic phototherapy. Biomater. Sci..

[59] Han X., Jing X., Yang D., Lin H., Wang Z., Ran H., Li P., Chen Y. (2018). Therapeutic mesopore construction on 2D Nb_2_C MXenes for targeted and enhanced chemo-photothermal cancer therapy in NIR-II biowindow. Theranostics.

[60] Dai C., Chen Y., Jing X., Xiang L., Yang D., Lin H., Liu Z., Han X., Wu R. (2017). Two-Dimensional Tantalum Carbide (MXenes) Composite Nanosheets for Multiple Imaging-Guided Photothermal Tumor Ablation. ACS Nano.

[61] Liu Z., Lin H., Zhao M., Dai C., Zhang S., Peng W., Chen Y. (2018). 2D Superparamagnetic Tantalum Carbide Composite MXenes for Efficient Breast-Cancer Theranostics. Theranostics.

[62] Yu X., Cai X., Cui H., Lee S.-W., Yu X.-F., Liu B. (2017). Fluorine-free preparation of titanium carbide MXene quantum dots with high near-infrared photothermal performances for cancer therapy. Nanoscale.

[63] Cao Y., Wu T., Zhang K., Meng X., Dai W., Wang D., Dong H., Zhang X. (2019). Engineered Exosome-Mediated Near-Infrared-II Region V_2_C Quantum Dot Delivery for Nucleus-Target Low-Temperature Photothermal Therapy. ACS Nano.

[64] Zada S., Dai W., Kai Z., Lu H., Meng X., Zhang Y., Cheng Y., Yan F., Fu P., Zhang X. (2020). Algae Extraction Controllable Delamination of Vanadium Carbide Nanosheets with Enhanced Near-Infrared Photothermal Performance. Angew. Chem. Int. Ed..

[65] Tang W., Dong Z., Zhang R., Yi X., Yang K., Jin M., Yuan C., Xiao Z., Liu Z., Cheng L. (2019). Multifunctional Two-Dimensional Core–Shell MXene@Gold Nanocomposites for Enhanced Photo–Radio Combined Therapy in the Second Biological Window. ACS Nano.

[66] Anasori B., Lukatskaya M.R., Gogotsi Y. (2017). 2D metal carbides and nitrides (MXenes) for energy storage. Nat. Rev. Mater..

[67] Assad H., Fatma I., Kumar A., Kaya S., Vo D.-V.N., Al-Gheethi A., Sharma A. (2022). An overview of MXene-Based nanomaterials and their potential applications towards hazardous pollutant adsorption. Chemosphere.

[68] Awasthi G.P., Maharjan B., Shrestha S., Bhattarai D.P., Yoon D., Park C.H., Kim C.S. (2020). Synthesis, characterizations, and biocompatibility evaluation of polycaprolactone–MXene electrospun fibers. Colloids Surf. A Physicochem. Eng. Asp..

[69] Carey M., Barsoum M.W. (2021). MXene polymer nanocomposites: A review. Mater. Today Adv..

[70] Fu B., Sun J., Wang C., Shang C., Xu L., Li J., Zhang H. (2021). MXenes: Synthesis, Optical Properties, and Applications in Ultrafast Photonics. Small.

[71] Fu Y., Zhang J., Lin H., Mo A. (2021). 2D titanium carbide(MXene) nanosheets and 1D hydroxyapatite nanowires into free standing nanocomposite membrane: In vitro and in vivo evaluations for bone regeneration. Mater. Sci. Eng. C.

[72] Soleymaniha M., Shahbazi M.-A., Rafieerad A.R., Maleki A., Amir A. (2019). Promoting Role of MXene Nanosheets in Biomedical Sciences: Therapeutic and Biosensing Innovations. Adv. Healthc. Mater..

[73] Xie Z., Chen S., Duo Y., Zhu Y., Fan T., Zou Q., Qu M., Lin Z., Zhao J., Li Y. (2019). Biocompatible Two-Dimensional Titanium Nanosheets for Multimodal Imaging-Guided Cancer Theranostics. ACS Appl. Mater. Interfaces.

[74] Iravani P., Iravani S., Varma R.S. (2022). MXene-Chitosan Composites and Their Biomedical Potentials. Micromachines.

[75] Jamalipour Soufi G., Iravani P., Hekmatnia A., Mostafavi E., Khatami M., Iravani S. (2022). MXenes and MXene-based Materials with Cancer Diagnostic Applications: Challenges and Opportunities. Comments Inorg. Chem..

[76] Mostafavi E., Iravani S. (2022). MXene-Graphene Composites: A Perspective on Biomedical Potentials. Nano-Micro Lett..

[77] Zhou B., Yin H., Dong C., Sun L., Feng W., Pu Y., Han X., Li X., Du D., Xu H. (2021). Biodegradable and Excretable 2D W1.33C i-MXene with Vacancy Ordering for Theory-Oriented Cancer Nanotheranostics in Near-Infrared Biowindow. Adv. Sci..

[78] Gazzi A., Fusco L., Khan A., Bedognetti D., Zavan B., Vitale F., Yilmazer A., Delogu L.G. (2019). Photodynamic Therapy Based on Graphene and MXene in Cancer Theranostics. Front. Bioeng. Biotechnol..

[79] Wang Y., Feng W., Chen Y. (2020). Chemistry of two-dimensional MXene nanosheets in theranostic nanomedicine. Chin. Chem. Lett..

[80] Sivasankarapillai V.S., Somakumar A.K., Joseph J., Nikazar S., Rahdar A., Kyzas G. (2020). Cancer theranostic applications of MXene nanomaterials: Recent updates. Nano-Struct. Nano-Objects.

[81] Iravani S., Varma R.S. (2022). MXenes in photomedicine: Advances and prospects. Chem. Commun..

[82] Liu Z., Zhao M., Yu L., Peng W., Chen Y., Zhang S. (2022). Redox chemistry-enabled stepwise surface dual nanoparticle engineering of 2D MXenes for tumor-sensitive T1 and T2 MRI-guided photonic breast-cancer hyperthermia in the NIR-II biowindow. Biomater. Sci..

[83] Liu G., Zou J., Tang Q., Yang X., Zhang Y.-W., Zhang Q., Huang W., Chen P., Shao J., Dong X. (2017). Surface Modified Ti_3_C_2_ MXene Nanosheets for Tumor Targeting Photothermal/Photodynamic/Chemo Synergistic Therapy. ACS Appl. Mater. Interfaces.

[84] Iravani S., Varma R.S. (2022). Smart MXene quantum dot-based nanosystems for biomedical applications. Nanomaterials.

[85] Shao J., Zhang J., Jiang C., Lin J., Huang P. (2020). Biodegradable titanium nitride MXene quantum dots for cancer phototheranostics in NIR-I/II biowindows. Chem. Eng. J..

[86] Lim G.P., Soon C.F., Ma N.L., Morsin M., Nayan N., Ahmad M.K., Tee K.S. (2021). Cytotoxicity of MXene-based nanomaterials for biomedical applications: A mini review. Environ. Res..

[87] Johnson K.K., Koshy P., Yang J.-L., Sorrell C.C. (2021). Preclinical Cancer Theranostics-From Nanomaterials to Clinic: The Missing Link. Adv. Funct. Mater..

[88] Lin H., Gao S., Dai C., Chen Y., Shi J. (2017). A two-dimensional biodegradable niobium carbide (MXene) for photothermal tumor eradication in NIR-I and NIR-II biowindows. J. Am. Chem. Soc..

[89] Zhao X., Wang L.-Y., Li J.-M., Peng L.-M., Tang C.-Y., Zha X.-J., Ke K., Yang M.-B., Su B.-H., Yang W. (2021). Redox-Mediated Artificial Non-Enzymatic Antioxidant MXene Nanoplatforms for Acute Kidney Injury Alleviation. Adv. Sci..

[90] Liang R., Li Y., Huo M., Lin H., Chen Y. (2019). Triggering Sequential Catalytic Fenton Reaction on 2D MXenes for Hyperthermia-Augmented Synergistic Nanocatalytic Cancer Therapy. ACS Appl. Mater. Interfaces.

